# Minithoracotomy for Aortic Valve Replacement: Are Perioperative Advantages Consistent Across Settings?

**DOI:** 10.1093/icvts/ivag151

**Published:** 2026-05-16

**Authors:** Bedirhan Buğra Bayıcı, Fatih Kızılyel

**Affiliations:** Department of Cardiovascular Surgery, Koşuyolu High Specialization Training and Research Hospital, Istanbul, 34846, Turkey; Department of Cardiovascular Surgery, Koşuyolu High Specialization Training and Research Hospital, Istanbul, 34846, Turkey

Dear Editor,

We read with great interest the recent article by Ríos-Ortega *et al.*^1^ which compares minithoracotomy (MT) and full sternotomy (FS) approaches for aortic valve replacement. The authors should be commended for providing data from a Latin American cohort and for strengthening their analysis through propensity score matching.

The observation that early and mid-term mortality rates were comparable between the 2 approaches is clinically reassuring and aligns well with previously published data.^2^^,^^3^ At the same time, a few points deserve further discussion.

The higher rate of postoperative atrial fibrillation (POAF) in the MT group is particularly noteworthy. The authors suggest that their pericardial drain positioning—specifically, contact with right-sided cardiac structures—may have contributed to this finding, which is a reasonable explanation. Still, it would be interesting to know whether other factors, such as longer operative times, the degree of pericardial manipulation, or even the learning curve associated with the technique, might also have played a role.

In addition, cardiopulmonary bypass and cross-clamp times remained longer in the MT group even after matching. While minimally invasive approaches are often associated with less surgical trauma and potentially faster recovery, longer operative durations could partly offset these advantages.^3^^,^^4^^,^^5^ A more detailed discussion of how these time differences may have influenced postoperative outcomes would add further clarity.

Another point worth highlighting is the lack of difference in intensive care unit and overall hospital stay. The authors appropriately note that this may be related to institutional and logistical factors, including the absence of an enhanced recovery after surgery (ERAS) programme and the fact that many patients come from distant regions. In this context, it would be valuable to know whether structured fast-track or ERAS pathways are being considered, as these could help patients benefit more fully from minimally invasive strategies.

Overall, this is a well-conducted and valuable study. Further clarification of these aspects would, in our view, help to better contextualize the findings and support optimal patient selection and perioperative management in minimally invasive AVR.

## Data Availability

Not applicable.
